# Can biosampling really be “non-invasive”? An examination of the socially invasive nature of physically non-invasive biosampling in urban and rural Malawi

**DOI:** 10.1080/11287462.2024.2398303

**Published:** 2024-09-09

**Authors:** Myness Kasanda Ndambo, Christopher Bunn, Martyn Pickersgill, Robert C. Stewart, Amelia C. Crampin, Maisha Nyasulu, Beatson Kanyenda, Wisdom Mnthali, Eric Umar, Rebecca M. Reynolds, Lucinda Manda-Taylor

**Affiliations:** a Malawi Epidemiology and Intervention Research Unit, Lilongwe, Malawi; b School of Social and Political Sciences, School of Health and Wellbeing, University of Glasgow, Glasgow, UK; c Centre for Biomedicine, Self and Society, Usher Institute, University of Edinburgh, Edinburgh, UK; d Division of Psychiatry, College of Medicine and Veterinary Medicine, University of Edinburgh, Edinburgh, UK; e School of Global and Public Health, Kamuzu University of Health Sciences; f School of Health and Wellbeing, University of Glasgow, Glasgow, UK; g Epidemiology and Population Health, London School of Hygiene and Tropical Medicine, London, UK; h Malawi Epidemiology and Intervention Research Unit, Karonga, Malawi; i Centre for Cardiovascular Science, Queen’s Medical Research Institute, University of Edinburgh, Edinburgh, UK

**Keywords:** Biomarkers, biosampling, glucocorticoids, non-invasive, therapeutic misconception

## Abstract

Glucocorticoids are understood to represent useful biomarkers of stress and can be measured in saliva, hair, and breastmilk. The collection of such biosamples is increasingly included in biobank and cohort studies. While collection is considered “non-invasive” by biomedical researchers (compared to sampling blood), community perspectives may differ. This cross-sectional, qualitative study utilising eight focus groups aimed to determine the feasibility and acceptability of collecting ostensibly “non-invasive” biological samples in Malawi. Breastfeeding women, couples, field workers, and healthcare providers were purposively sampled. Data about prior understandings of, barriers to, and feasibility of “non-invasive” biosampling were analysed. Participants described biomaterials intended for “non-invasive” collection as sometimes highly sensitive, with sampling procedures raising community concerns. Sampling methods framed as *physically* “non-invasive” within biomedicine can consequently be considered *socially* “invasive” by prospective sample donors. Biomedical and community framings of “invasiveness’ can therefore diverge, and the former must respond to and be informed by the perspectives of the latter. Further, considerations of collection procedures are shaped by therapeutic misconceptions about the immediate health-related utility of biomedical and public health research. When researchers engage with communities about biosampling, they must ensure they are not furthering therapeutic misconceptions and actively seek to dispel these.

## Introduction

The detrimental effects of stress and mental health conditions occurring in the antenatal period, such as depression and anxiety, are widely regarded as significant public health problems globally (Agongo et al., 2021; Ahram et al., 2014; Appelbaum et al., 1982; Ashforth, 2014). Studies from low – and middle-income countries (LMICs) have reported that antenatal depression – for which environmental stressors, including poverty, play an aetiological role – may be a risk factor for impaired fetal growth and poorer neonatal outcomes (Agongo et al., 2021; Ahram et al., 2014; Appelbaum et al., 1982; Ashforth, 2014; Barchi & Little, 2016; Bertolt, 2018; Bitta, 2022). Glucocorticoids are commonly used as a stress biomarker and are increasingly analysed within biomedical and public health research, including in biobanking and cohort studies in LMICs.

Glucocorticoids are generally extracted through biosampling of saliva, hair, and breastmilk (Bleker et al., 2019). These are widely regarded by biomedical and population health researchers as “non-invasive” methods of obtaining human biomaterials (e.g. for biobanks) since they do not involve skin puncture (unlike blood collection). Sampling of biomaterials can be controversial; in Malawi (and elsewhere in Africa), for instance, there can be concerns about what researchers will do with blood samples following collection (Braun & Clarke, 2006; Broekstra et al., 2020; Burke, 2014; Burnard et al., 2008), with the real risk of exploitation demanding ongoing consideration (Christoffels & Abayomi, 2020; Coetzee et al., 2012; Compaoré et al., 2018). Prior research by ourselves and others has also documented concerns relating to witchcraft and vampirism (Crampin et al., 2012; Creswell, 2013; de Vries et al., 2015, 2017).

While “non-invasive” methods can be framed within biomedicine as eliding these concerns, perspectives of “invasiveness’ within communities may vary – relating to different perspectives on bodies and bodily materials between researchers and participants (Glover, 2014). An example of hair is illustrative. Within Africa, hair and hair care are laden with gendered cultural meanings (Herbertson et al., 2021; Kaler, 2009). In some African contexts, the sampling of hair is also associated with concerns relating to witchcraft and the potential harm that could be experienced by donors as a consequence (Kapumba et al., 2020; Kapumba et al., 2022; Kekwaletswe et al., 2018; Kimmelman, 2007).

Community concerns can substantiate or catalyse broader fear and distrust about biomedical and population health research. Negative impacts include a reduced likelihood of potential participants consenting to participate in studies (Lazovski et al., 2009). Community engagement is often presented as a “solution” to that “problem”; however, under this framing, engagement can focus on providing technical information rather than promoting dialogue (MacIntyre et al., 2013; Manda-Taylor et al., 2019; Manda-Taylor et al., 2021). Population health studies in Malawi that some of the author team are involved in explicitly work with communities to address such concerns as part of ongoing dialogical activities, including through tours of laboratory and sample storage facilities for community leaders and people of influence.

Communities participating within biomedical and population health research can sometimes anticipate that research results will be delivered back to them; yet, this does not always happen – with implications for trust and researcher credibility (Burnard et al., 2008). Further, participants can have expectations about the knowledge generated that might not align with those of the researchers themselves; for instance, the receipt of individualised, health-related results (Morgan, 2012). While some diagnostic or therapeutic practices might be provided as part of research within LMICs, this is not always the aim of research and is not always available (despite ethical imperatives to maximise care provision for participants) (Mtunthama et al., 2021; Mungwira et al., 2015). Consequently, therapeutic misconceptions during participation are feasible (Munung et al., 2016; Muula & Mfutso-Bengo, 2007; National Statistical Office, 2021). They are perhaps especially likely when an act akin to a biomedical procedure – such as donating a human biomaterial – takes place.

Reduced participation in African studies involving biosampling could have epistemic and, ultimately, clinical implications due to the unequal representation of samples internationally. Global participation in biobanks is already dominated by white, Western-dwelling populations, with knock-on consequences for equitable research-led health innovations (Masiye et al., 2023). This is in the context of other barriers concerning participation in biomedical and population health research (Crampin et al., 2012; Matandika et al., 2020; Mbililishaka, 2018; Mezinska et al., 2020). Notable among these are the issues mentioned above of participant trust, alongside researcher trustworthiness (Creswell, 2013; Mfutso-Bengo et al., 2008; Mfutso-Bengo et al., 2015).

Generation Malawi is a multi-generational family/birth cohort established to study the longitudinal course of long-term mental and physical health conditions in Malawi and pregnancy, early life, and intergenerational effects on these trajectories. It is funded by the Wellcome Trust and the UK Medical Research Council (MRC) and implemented by the Malawi Epidemiology and Intervention Research Unit (MEIRU) (an international non-governmental research organisation). The objectives of Generation Malawi include the establishment of a biorepository, including biosamples of saliva, hair, and breastmilk for analysis of glucocorticoids. The regulatory landscape for biosampling within Malawi and sub-Saharan Africa continues to shift and change. However, sample collection for specific research – following appropriate informed consent – has been permitted for some time (Burnard et al., 2008; NCDI Poverty Network. The Malawi NCDI Poverty Commission Report, 2018; Ndambo et al., 2023). The study reported here aimed to investigate community perspectives around sampling of saliva, hair, and breastmilk to (1) examine prior understandings and experiences of “non-invasive” biosampling in the communities anticipated to participate in Generation Malawi, (2) explore barriers to “non-invasive biosampling”, and (3) determine the feasibility of biosampling as part of Generation Malawi. Through this study, we intended to generate data that would be relevant and applicable to other research programmes that seek to employ “non-invasive” methods of biosampling to enhance their ethicality and acceptability.

## Methods

We conducted a formative, cross-sectional qualitative study employing focus group discussions (FGDs) with breastfeeding women, couples (women and their male partners), health care providers (HCPs), and research field workers to explore the feasibility and acceptability of collecting biosamples regarded as “non-invasive” by biomedical researchers (breastmilk, hair, and saliva) from mothers, fathers, and infants.

### Study setting

Malawi is an eastern sub-Saharan African country bordered by Tanzania, Mozambique and Zambia. It is a low-income country with a rapidly growing population of around 19.9 million and a life expectancy of 64 years (Nderitu & Emerson, 2024). Most of Malawi’s population lives in rural areas where 70% of people live in “multidimensional poverty” (Ng’oma et al., 2019). Malawians have access to an “Essential Healthcare Package” through government facilities. However, this remains limited in scope, with a spend of just USD34 per capita in 2020/21 (Niehaus, 2002) and significant gaps between pharmaceutical needs and availability (Nwogu et al., 2019).

The study was conducted at two sites in Malawi where Generation Malawi operates: Area 25 in Lilongwe and Chilumba in Karonga District. Situated within the Central Region of Malawi, Lilongwe is the capital city of Malawi, with a high rate of urbanisation. Chilumba is a small town in the Northern Region of Malawi, located along the western shores of Lake Malawi in the rural Karonga District. The Chewa ethnic group reside predominantly in the Central Region, while the Tumbuka ethnic group live predominantly in the Northern Region. Nonetheless, both areas are home to people who identify with various ethnic groups.

MEIRU is a research institution with research sites in Lilongwe and Chilumba. It has been undertaking large-scale, long-term, population-based studies in Chilumba for several decades and in Lilongwe since 2013. Funding for its activities primarily comes from UK-based sponsors (e.g. Medical Research Council, National Institute for Health Research, Wellcome). It has an active programme of community engagement.

### Study population

Given the differences between Lilongwe and Chilumba, our FGDs included perspectives from wide-ranging socio-economic and cultural backgrounds. The Lilongwe research site hosts a diverse population, with many rural-urban migrants from various regional and tribal backgrounds and monetised economic activity. By contrast, the Chilumba site has greater homogeneity, and its economy relies heavily on subsistence activities. Our research sought to capture diverse and varied viewpoints on the feasibility and acceptability of collecting biosamples in the populations where research staff would invite community members to participate in Generation Malawi.

The study population included community members (breastfeeding women and couples) in the MEIRU sites in Lilongwe and Karonga, research field workers, and HCPs from these districts who served the Generation Malawi research sites or nearby health facilities (Area 25 Health Centre in Lilongwe City and Chilumba Rural Hospital in Karonga District). Despite the diversity of participants, we found key overlaps in their contributions to the FGDs, which we focus on below.

Participants were purposively sampled from a database of people who participated in previous MEIRU research and consented to be contacted about future research (Omotoso, 2018). Purposive sampling enabled the identification of individuals whose characteristics would be similar to those of prospective donors and sample collectors in establishing a biorepository and targeting the recruitment of participants who would most likely be available and willing to discuss sensitive topics. In particular, our decision to only engage breastfeeding and not pregnant women was related to the aims of the population health research with which our study was associated to collect hair, saliva, and breastmilk samples from breastfeeding women.

### Data collection procedures

Over November/December 2021, eight FGDs were conducted in Lilongwe and Chilumba. The first author (MKN) called prospective participants to introduce the study. Participants who volunteered were recruited and invited to participate in an FGD the following day. Four FGDs were conducted per site in Karonga and Lilongwe. One FGD at each site was conducted with HCPs, field workers, breastfeeding women, and couples (i.e. one man and one woman). The resonant data between the FGDs suggests that additional FGDs would not have produced markedly different data of analytic salience.

78 potential participants were invited to participate in this study. All but four HCPs agreed to take part; those who declined cited busy schedules. Two spouses in Karonga who had elected to participate in the couple's FGDs did not arrive as planned. In Lilongwe, three spouses did not participate in the couple's FGDs, and one breastfeeding woman withdrew because her baby was distressed. While these eventualities led to some unintended shifts in focus group composition, all contributions were retained to preserve the co-constructed nature of the data generated (Peeters et al., 2014). All invited field workers accepted to participate. The total sample size was thus *N* = 68, with 6–12 participants per FGD (Table 1).

**Table 1.** Focus groups conducted.

	Urban Lilongwe		Rural Karonga	
	Male	Female	Male	Female
Breastfeeding mothers	–	9	–	10
Couples	2	5	7	5
Health care providers	1	7	4	3
Field workers	4	3	7	1
Total	7	24	18	19

Participant information sheets and informed consent forms were developed in English and translated into Chichewa (the national language and predominant local language in the Central District) and Chitumbuka (the dominant local language in the Karonga District). MKN is a native Tumbuka and Chichewa speaker who translated them into Chichewa and Tumbuka.

As part of obtaining informed consent, before each FGD began all participants were briefed about the study. Participants were assured their details would be omitted from transcripts to ensure confidentiality, and told that any information generated by the research might be published (but privacy would be maintained, and no personal details would be shared). They were reminded that their involvement in the study was voluntary and that withdrawal was permitted at any time without personal consequence. Participants were invited to ask questions, and asked for their written or thumb-printed consent.

A semi-structured FGD schedule was employed (supplementary file 1) to elicit rich data regarding their knowledge of ostensibly non-invasive sampling of hair, breastmilk, and saliva and their perspectives on the feasibility and acceptability of this for biomedical and population health research. Questions were, for instance, designed to elicit reflections relating to experiences of biosampling and the practicalities relating to this for Generation Malawi, as well as concerns – including community and individual perspectives on perceived risks and safeguarding measures of such a study.

As noted, the languages spoken in the FGDs were Chichewa in Lilongwe and Chitumbuka in Karonga. Like the consent forms, MKN initially developed the FGDs schedule in English and reviewed it with CB and LMT to ensure that the questions addressed the study objectives. Additionally, to further ensure fidelity, linguistic accuracy, and cultural appropriateness, MKN and CB back-translated the Chichewa and Tumbuka discussion guide into English to confirm that the intended meaning was accurately conveyed. Discussion guides were piloted with six MEIRU field workers and six Lilongwe and Karonga district community members to assess clarity, contextual relevance, comprehensiveness, and question flow. Questions that were identified as ambiguous were amended.

MKN, a social scientist with extensive experience conducting FGDs, convened the FGDs with three trained research assistants from MEIRU (co-authors BK, WM, and MN). MKN, BK, WM, and MN each led a separate FGD. The FGDs were conducted face-to-face yet physically distanced in compliance with COVID-19 mitigation protocols. They lasted approximately 60 minutes each. After each FGD, a debriefing session was conducted, summarising the discussion about the study objectives and addressing any concerns that arose before closing the session.

The study was approved by the Malawi National Health Sciences Research Committee (NHSRC) (protocol number 21/01/2653) and the College of Medical, Veterinary and Life Sciences Ethics Committee, University of Glasgow, UK (protocol number 200200082), and aligned with the Declaration of Helsinki (Phiri et al., 2018).

### Data analysis

Interviews were recorded, transcribed verbatim, and translated into English. WM and BK transcribed the Tumbuka discussions in Chilumba and MN for the Lilongwe discussions. As noted, all are native speakers fluent in their respective languages. MKN subsequently cross-checked and verified translations against the recordings.

Transcripts were anonymised, with each participant assigned a participant identity (ID) number. The data analysis undertaken in this study was primarily exploratory and inductive (Pickersgill, 2011), allowing for exploration and discovery within the data. It involved closely examining the data to identify patterns, themes, or insights pertinent to our research questions (Pickersgill, 2011). Following familiarisation, data were coded in MS Word, focusing on three domains of content: prior understandings of “non-invasive” sampling, barriers to collecting “non-invasive” samples, and feasibility of “non-invasive” sampling. The analysis was focused on understanding community perspectives and exploring barriers, followed by a broader consideration of unanticipated themes and how these could contribute to a more comprehensive understanding of the ethical and social dimensions of biomedical and public health research within Malawi. During this phase, MKN and co-author CB independently (to minimise researcher bias) recorded preliminary codes in the margins of the transcripts. Initial codes where then discussed, and a coding framework created (with differences reconciled through discussion) (Rwafa, 2016; Saeed et al., 2015). MKN then transferred all the English language transcripts into NVivo 12 for data management and applied the codebook to all transcripts. MKN and CB reviewed the coded dataset to identify patterns and develop content-orientated themes (Dunkel Schetter & Tanner, 2012). During this process, cross-tab reports were generated in Nvivo to compare responses between participants from Lilongwe and Karonga and across the different sub-groups included in the sample.

## Results

We report the findings of our analysis against the aims of our study, relating to prior understandings of, barriers to, and feasibility of “non-invasive” biosampling. The comparative aspects of our analysis did not identify any notable differences in responses from the two sites. Still, we identified differences in how women spoke about biosampling when participating in FGDs with other women compared to spouses. We report on these below.

### Prior understandings of “non-invasive” biosampling

We enquired about participants’ existing understandings of “non-invasive” biosampling and whether they had collected/provided such samples. HCPs and field workers were aware of sampling techniques for biomaterials like saliva, with HCPs more likely to be familiar with these:

Non-invasive sample collection procedure is when we collect samples without entering any device into the skin of a participant, and we collect it on the surface. P**5_HCP_KA**

Yes, we have done that before. It is the collection of samples without needle pricking or collecting blood or using anything inserted in the human body. P**7_Fieldworker_KA**

Despite familiarity with “non-invasive” biosampling, both HCPs and fieldworkers were somewhat skeptical about such practices given a lack of prior community familiarity:

The community has different views on different studies like the collection of saliva is not strange here, but fears will be added on hair and breastmilk since it's new since MEIRU started doing research activities. **P3_Fieldworker_KA**

HCPs and fieldworkers pointed to longstanding beliefs in *ufiti* [witchcraft] in the communities with which they worked:

Concerns will be there as regards our cultural beliefs. You can have a health talk, and after that, they agree to participate in the study, but if the child gets sick suddenly, the relatives will say, ‘It’s because of the sample of the hair which the researchers collected’. P**2_HCP_LL**

Concerns were, for instance, expressed that if participants experienced anything negative after submitting samples, HCPs and fieldworkers would have blame attributed to them and subsequently suffer consequences:

We live with the participants in the locations so that a child could be born with a problem, but if something bad happens, they might come to us and say it’s a result of the samples they submitted. This will be a big challenge, especially if the child dies. In the community, when a person is dead, there are many misconceptions about the cause of death surrounding witchcraft. Therefore, this is not a good approach in our country. P**6_Fieldworker_LL**

By contrast, no community members from either site had prior knowledge of “non-invasive” biosampling methods:

I have never heard about giving samples of saliva, breastmilk and hair, so people may not understand why you are collecting their hair since it’s new to them. **P3_Breastfeeding woman_KA**

Such sampling could be framed as “strange” if communities were insufficiently engaged about the procedures, resulting in participant disquiet and disinclination to participate:

This has never happened since we were born, and this would be a strange practice, and it will be difficult for a person to agree to participate. It is difficult because we already have misconceptions about the hair the barbers remove. We think they use it for rituals … there is a need for transparency to see how the processes are done, but if you go and collect samples, people will throw stones at you … . **P4_Man_LL_Couples**

Indeed, some participants felt that “non-invasive” biosampling was unacceptable:

[W]e have never heard this before, and this is an unusual practice for someone to collect hair, breastmilk and saliva. This is not true and acceptable in the community. P**5_Breastfeeding woman_LL**

Some people may not understand this study. It will be hard for people to give you their hair, saliva, and breastmilk because it is unusual here. **P7_Breastfeeding woman_KA**

These concerns do not differ significantly from the conflicts and tensions associated with biosampling techniques commonly understood within biomedicine as invasive; e.g. the sampling of blood. As one participant reminded us:

People in the study area have always associated the studies involving blood samples to the negative side, regarding us as blood sellers and satanic. Introducing this study involving hair, saliva and breastmilk, we will add fears, worries and negative attitudes that some people already have. **P1_ Fieldworker_KA**

As noted above (Braun & Clarke, 2006; Broekstra et al., 2020; Burke, 2014; Burnard et al., 2008), hair, saliva, and breastmilk hold cultural markers of value and meaning. Participants’ views of “non-invasive” biosampling suggest these techniques transgress social and cultural norms, breaking taboos – which can be understood as exogenous stressors. Consequently, communities might view it with fear. Non-invasive biosampling may not involve direct physical invasion, but nevertheless constitutes a social invasion by intruding on an individual's privacy and creating discomfort or vulnerability. It also stimulates concern around downstream physical consequences. The following section unpacks these fears in more detail.

### Barriers to “non-invasive” biosampling

In Malawi, using human biomaterials – especially blood – can be equated with leveraging these for *ufiti* (witchcraft) or vampirism (Shen et al., 2021). Our data serve as a reminder that the cultural meanings attached to other biomaterials that researchers might seek to collect – like saliva or hair – can also be linked to witchcraft (Singh et al., 2023; Staunton & Moodley, 2013), and which might act as a barrier for biosampling. Community members consequently voiced concerns about both participant and community safety from such “non-invasive” biosampling:

We believe that the powers of darkness use these things, and we think they want to use them for rituals, or they want to become rich using these things like saliva […] Some samples are used to cause mental disorder, and they make a person go mad. **P2_Breastfeeding woman_KA**

Removing hair, for example, is part of a cleansing process undertaken by someone as part of rituals conducted after an initiation ceremony, after a funeral ceremony, and after a baby’s umbilical cord has detached (Staunton & Moodley, 2013). Such hair is removed and disposed of to avoid others accessing it with evil intentions:

When girls have experienced first menstruation, they go under counselling, and after that, they cut off their hair. Some people cut off the hair soon after the funeral burial to chase away demons or death spirits, so they use it in special ceremonies. **P1_Field worker_LL**

In our culture, it is said that after we cut the baby’s hair, we should throw it in the pit latrine, so it would be suspicious for a strip of the baby’s hair to be taken and put somewhere; this would also be difficult for us to explain to older women that they want to use the hair as a sample. It will be difficult for them to understand, and they may threaten us, and we might also receive different kinds of misconception from different people who will frighten us. **P4_Breastfeeding woman_LL**

Participants were surprised to hear that breastmilk could have scientific value. They reported that it is believed that breastmilk should only be used to feed a baby since expressing it manually is a taboo which may result in harm to a child:

People believe that when a parent pours her breastmilk on the ground, the child won’t grow or may face some misfortunes. We believe that breastmilk is for an infant feeding only … this is a taboo … **P3_Fieldworker_LL**

People believe that one can get rich once he/she gets breastmilk from the mother who has just given birth or the placenta or the breast itself … it is also believed that breastmilk can cure snake venom … **P4_Fieldworker_KA**

… people will be scared seeing someone pressing breastmilk and giving it to someone … because these are the things other people use in evil ways. **P2_ Breastfeeding woman_LL**

Concerns were also raised regarding saliva sample collection:

Strange things happen in this world. What if I submit saliva today, and after a week, I begin to notice some changes in my mouth, such as a feeling of a heavy tongue or becoming mute? Saliva is sensitive and could be used to kill the person who has submitted it. **P1_Breastfeeding woman_LL.**

Participants also explained that saliva is regarded as a neutraliser; when applied where someone intends to use the other person’s body fluids, the power of *ufiti* is neutralised:

People can use saliva to kill someone through witchcraft. Another example is that if one wants to use your urine for rituals, once you spit where you have urinated, everything involving rituals is neutralised. Meaning no one can use your urine for rituals anymore. This makes this study very sensitive, and people will fear to participate in this study. **P5_Fieldworker_KA**

Participants also explained that “non-invasive” biosample collection might introduce tension between spouses and between them and their relatives or neighbours since people might have differing views about research participation. This could lead to accusations being made toward those consenting to take part, particularly women. These concerns came up in all the FGDs except for the couple’s groups where both the husband and wife were present:

[F]athers would find it difficult to understand because they do not usually go to the Health Centres. They sometimes go to the hospital when they are in a critical condition. It takes time for them to understand things, and maybe two out of 10 men would understand and accept. **P3_HCP_KA**

We may understand, but our spouses at home may not understand because other men are tough, and they can say, ‘I don’t want to hear this again’. **P6_Breastfeeding woman_LL**

The absence of accounts of the potential for “non-invasive” biosampling to cause spousal tensions in the couple's focus groups reinforces these concerns.

The data suggest two overarching barriers to collecting “non-invasive” biosamples: first, the social and cultural significance of hair, breastmilk, and saliva; second, patriarchal norms, which might mitigate against sample collection within heterosexual couples even when the female partner consents. Accordingly, participants reported that prima facie collecting hair, saliva, and breastmilk samples for biomedical and public health research would not be acceptable. However, as we will see below, they also reflected that various mechanisms might mitigate concerns.

### Feasibility of “non-invasive” biosampling

Despite cultural concerns, conflicts, and tensions with biosampling saliva, hair, and breastmilk, we sought participants’ views about whether such sample collection might still be possible. Our participants reported various means and criteria through which studies utilising this practice might be undertaken – including intensive community engagement:

Discuss with the community leaders first […] if you go by yourself, people will find it difficult to trust you with their breastmilk or infant’s hair, and they won’t participate. You should also involve HSAs and volunteers for them to trust you. **P6_HCP_LL**

As part of community engagement, the importance of detailed information provision was highlighted:

Information about this research has to be explained to everyone more clearly, like the connection between hair, saliva, breastmilk and depression, because many questions will be raised. **P2_Fieldworker_ LL**

Some participants reflected that they would prefer to see the procedures conducted on someone else (i.e. a researcher) before donating biomaterials:

When you want to collect these samples, you should try this on yourselves so that we will see and believe it. Yes, because if you go mad, it is bad, but if nothing bad has happened, it is just okay. **P5_Breastfeeding woman_KA**

From the FGDs in Karonga, some participants believed that MEIRU had brought benefits through previous studies. Participants felt that attempts to encourage donation of hair, saliva, and breastmilk might succeed because of their prior research experience and participation with MEIRU, which was framed as benefiting the entire community:

I think the community will welcome this study because MEIRU has conducted several studies with the same people on different research studies for a long time, and people have benefitted differently. **P2_Woman_KA_Couples**

Further, the provision of feedback to participants following sample analysis was described as a means through which to build trust in the use of biomaterials:

This will depend on the results. When you take the first samples and bring the results to the participants, people will continue with you, but when you don’t, people will be suspicious. **P3_Woman_LL_Couples**

Alongside providing a sample, some noted that research participation could lead to potential and swift health-related benefits:

It is acceptable and important because we will know if we have the disease so that the experts can help us accordingly. **P1_Breastfeeding woman _KA**

I think people in our community will understand and welcome it since we are the beneficiaries. For example, if one is found with a certain disease, they ensure the person is helped through medical care. So I think people will be happy with this and participate in this study. **P6_Man_KA_Couples**

Such comments illustrate a therapeutic misconception about the kinds of research often conducted using biological samples (including Generation Malawi) (Munung et al., 2016; Stewart, 2007): these are unlikely to afford immediate health-related benefits to participants, including confirming the presence/absence of disease. Given that sample collection is a frequent part of clinical care (e.g. blood tests), this therapeutic misconception – arising from unfamiliarity with the aims of public health research in general and, more specifically, the collection of non-invasive samples – is unsurprising. It requires careful addressing to ensure participants gain a fuller understanding of the nature of research not designed to confer immediate benefits.

## Discussion

**Figure 1.**
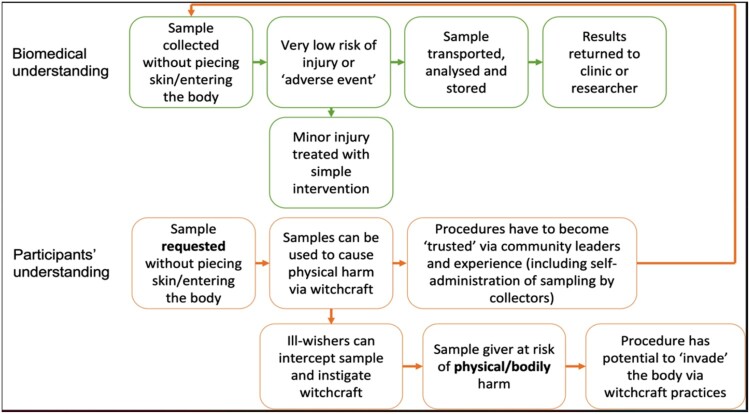
Divergent biomedical and community perspectives on biosampling.

The feasibility and acceptability of collecting non-invasive biosamples (such as hair, breastmilk, and saliva) in Malawi were investigated through a cross-sectional, focus group study. Our study underscored that many people strongly believe in *ufiti* (witchcraft) and revealed how these beliefs raise concerns about “non-invasive” biosampling. This finding resonates with previous research, including our own, which has illustrated concerns about taking blood samples given the association of this practice with vampirism, witchcraft, and selling blood (Crampin et al., 2012; Creswell, 2013). These concerns indicate a lack of trust in researchers, which stems from historical and problematic practices in medical research in sub-Saharan Africa (Stewart et al., 2019). These can be regarded as sitting alongside ongoing concerns about participant exploitation (Masiye et al., 2023; Subramani & Biller-Andorno, 2022). Given the sensitive nature of biomaterials collected “non-invasively”, collection practices can be considered socially invasive. This is illustrated in Figure 1 below:

While concerns about *ufiti* were reported in all FGDs, only women, fieldworkers and HCPs raised the issue of spousal power dynamics, suggesting that husbands and other male figures were sources of likely objection to “non-invasive” biosampling. Our participants’ accounts align with literature that describes male domination over women’s bodies across a range of African cultures (Thong et al., 2016) and how this is entangled with colonialism (Tindana et al., 2015) and medical imperialism (Matandika et al., 2020; Mezinska et al., 2020; National Statistical Office, 2021)

Despite participant concerns, we found that sampling hair, saliva, and breastmilk might nevertheless be feasible – from our respondents’ perspective – if close attention is paid to community engagement. In part, this reflects the positive experiences of our participants with the principal research organisation hosting the Generation Malawi programme – MEIRU – through previous studies. Our findings resonate with other work assessing the feasibility of collecting biosamples, where participation in research was likewise motivated by prior positive experiences with researchers (Tindana et al., 2020). This implies evidence of trust and solidarity between communities within MEIRU sites, HCPs, and researchers. It also demonstrates that trust in researchers and open communication could profoundly influence decision-making and acceptability, especially when introducing new and potentially sensitive research procedures (Creswell, 2013; Mfutso-Bengo et al., 2008; Mfutso-Bengo et al., 2015). This increases yet further researchers’ responsibilities to enact and demonstrate trustworthiness.

Participant trust in research and researchers might override concerns about the taboo nature of donating biosamples. However, this raises ethical concerns when that trust is partly founded on therapeutic misconceptions. We found that several participants cited the anticipated health-related benefits of participation as a reason for consenting to biosampling. Our results align with other studies which demonstrated that participants in biomedical and public health research were motivated to submit samples because they wanted to know about their health (Tindana et al., 2020; Tong et al., 2007). Yet, the temporalities of research like Generation Malawi might not align with the expectations of participants, nor might the extent and nature of any health-related benefits that might be afforded. As part of the principle of beneficence, researchers must work hard to maximise the benefits of research for participants. At the same time, the discussion of these benefits must be balanced and proportionate; otherwise, participant autonomy is compromised.

It is vital that participants, when consenting to take part in biomedical and public health research, are enabled to understand the realistic scope and limits of this (Crampin et al., 2012; Varkey, 2021; UNICEF Malawi Health, 2020/21). This is particularly the case when donating biomaterials that might be presumed to be physically “non-invasive” biosampling, given their socially invasive nature. Accordingly, we recommend that community engagement activities should not merely aim to “reassure” communities but actively strive to enhance autonomy (Wilkins, 2018) – as a key rationale for informed consent (World Bank Group, 2020; World Medical A, 2001) – through frank conversations about the scope, limits and possibilities of research. We argue that this contributes to demonstrating trustworthiness, as opposed to standard assurances that trust is merited (Wilkins, 2018). Ultimately, this may enhance the likelihood of participation in biosampling; however, this cannot be taken for granted, nor should it be the primary goal of engagement. Future work would usefully examine in greater granularity what health-related benefits participants assume will emerge from research programmes like Generation Malawi to underpin engagement activities of the kind we recommend.

## Study strengths and limitations

Drawing participants from previous research was a limitation in our study, as it could have resulted in particular tropes being foregrounded in the FGDs (e.g. around the value of community engagement and population health research) based on prior, positive experiences with MEIRU (which themselves could have been over-represented). The absence of some male spouses from the couples’ FGDs is a limitation and presents a practical and analytical dilemma. At the time of data collection, researchers opted for an inclusive approach, deciding not to turn away anyone who had given their time to participate. When analysing the data, we regarded our FGD data as co-constructed through interaction (Peeters et al., 2014), and removing contributions from those attending without spouses would limit our ability to make sense of the interactions through which the data were generated. Finally, we made a deliberate but potentially limiting choice not to foreground perspectives on the “non-invasive” biosampling of breastfeeding women. Instead, we have purposely presented views from across the social constellation through which such sampling procedures would be performed, recognising that individualising women’s responses would artificially separate them from the (power-laden) relationships in which they are enmeshed.

However, this study has strengths too, aside from the empirical and conceptual contribution it makes. It was conducted in two districts with different cultural backgrounds in Malawi with 68 participants, which ensured a broad range of perspectives were generated and analysed. The study was also performed in the communities where Generation Malawi will occur, providing reliable data to inform future biomedical and population health research.

## Conclusion

This cross-sectional study sought to assess the feasibility and acceptability of collecting ostensibly physically non-invasive biomaterials for glucocorticoid analysis (specifically, hair, saliva, and breastmilk) in Malawi. Such practices could be acceptable, partly due to existing trust in the research organisation undertaking biosampling and partly due to therapeutic misconceptions about the health-related benefits that participation could afford. However, the samples in question are highly sensitive, for instance, due to their association with witchcraft. Accordingly, even though these samples are widely perceived in biomedicine and public health as physically “non-invasive”, they are considered socially invasive by potential participants in Malawi – and viewed as potentially leading to physical harm via practices of witchcraft. While those who participated in our study underscored the importance of community engagement, this must proceed to enhance autonomy through realistic appraisals of the scope and timelines of any health-related benefits that participants might be afforded. In so doing, the trustworthiness of research and researchers might also be enhanced. In sum, we argue that strengthening public understanding of the research process, to diminish therapeutic misconceptions and demonstrate trustworthiness, is the only ethical means of breaking down barriers to sample collection (Crampin et al., 2012; UNICEF Malawi Health, 2020; Varkey, 2021). Simply persuading individuals to donate samples because they might believe this will confer swift health-related benefits does nothing to enhance autonomy. Doing so would, in effect, be unethically misleading participants through deliberate refusal to modify a salient misunderstanding.

## Data Availability

The datasets generated during and analysed during the current study are available from the corresponding author upon reasonable request and subject to any relevant ethical and governance approvals.
